# Two Brief Steps, Better Foresight: Cognitive Screening and Adverse Outcomes in Older Adults Admitted From the Emergency Department

**DOI:** 10.1111/jgs.70378

**Published:** 2026-04-10

**Authors:** Gabriel Stanziola de Moraes, Thiago Junqueira Avelino-Silva, Kenneth E. Covinsky, Christopher R. Carpenter, Mfon E. Umoh, Christian V. Morinaga, Pedro K. Curiati, Márlon Juliano Romero Aliberti

**Affiliations:** 1 Geriatric Emergency Department Research Group (ProAGE), Hospital Sírio-Libanês, São Paulo, São Paulo, Brazil; 2 Laboratorio de Investigaçao Medica Em Envelhecimento (LIM-66), Servico de Geriatria, Hospital das Clinicas HCFMUSP, Faculdade de Medicina, Universidade de Sao Paulo, Brazil; 3 Division of Geriatrics, Department of Medicine, University of California San Francisco, San Francisco, California, USA; 4 Department of Emergency Medicine, Mayo Clinic, Rochester, Minnesota, USA; 5 Division of Geriatric Medicine and Gerontology, Department of Medicine, Johns Hopkins School of Medicine, Baltimore, Maryland, USA

**Keywords:** activities of daily living, acute care, cognitive impairment, delirium, risk stratification

## Abstract

**Background:**

Delirium predicts adverse outcomes in older emergency department (ED) patients, but many acutely ill patients without delirium have underlying cognitive impairment that goes unrecognized. Whether cognitive impairment screening improves risk prediction beyond delirium remains uncertain. We compared practical bedside screening strategies for delirium, cognitive impairment, or both for predicting 90-day functional decline and mortality in older adults admitted from the ED.

**Methods:**

A prospective cohort comprising patients aged ≥ 65 years admitted from the ED of a large hospital in São Paulo, Brazil. Trained professionals screened for delirium using the brief Confusion Assessment Method (bCAM) and for cognitive impairment using the 10-point Cognitive Screener (10-CS). Patients were classified as having normal cognition (bCAM negative, 10-CS > 5), delirium (bCAM positive), or cognitive impairment without delirium (bCAM negative, 10-CS ≤ 5). Blinded investigators assessed decline in basic activities of daily living (ADL) and mortality within 90 days of admission. Fine-Gray models (death as a competing risk) and Cox models estimated associations with outcomes, adjusting for sociodemographic and clinical factors.

**Results:**

Among 830 patients (mean age = 80 ± 9 years; women = 47%), 427 (51.5%) had normal cognition, 171 (20.6%) had delirium, and 232 (27.9%) had cognitive impairment without delirium. Among delirium-negative patients with cognitive impairment, 52% had no documented dementia diagnosis or reported memory problems. Compared with normal cognition, cognitive impairment without delirium was associated with 90-day functional ADL decline (sub-HR = 1.60; 95% CI = 1.03–2.49) and mortality (HR = 2.31; 95% CI = 1.18–4.51), with risks similar to those observed in delirium. A staged strategy (bCAM first, then 10-CS if bCAM negative) showed higher discrimination than delirium-only or 10-CS-only screening.

**Conclusions:**

Cognitive impairment without delirium is common, often unrecognized, and predicts 90-day adverse outcomes in older patients admitted from the ED. A brief staged screening strategy integrating delirium assessment with cognitive impairment testing among delirium-negative patients may enhance early detection of cognitive vulnerability and support care planning.

## 1 ∣ Introduction

Population aging has increased emergency department (ED) utilization by older adults worldwide. In the United States, older adults account for one in five ED visits, totaling approximately 29 million visits annually, and in low- and middle-income countries, they now represent about one-quarter of adult ED visits [1, 2]. Delirium occurs in up to 20% of older adults admitted from the ED [3]. Dementia is present in about 9% to 25% of older adults admitted from the ED [4]. When delirium, dementia, and milder cognitive deficits are considered together, cognitive impairment affects nearly 40% of admissions [4, 5]. Cognitive impairment increases the risk of diagnostic delays, adverse events, prolonged hospitalization, and death [6, 7]. However, cognitive impairment remains unrecognized in up to 70% of older ED patients [5, 8].

Clinical guidelines recommend screening for delirium and cognitive impairment in the ED to inform care and risk stratification [9], but routine screening remains uncommon [10-12]. Barriers include limited workflow integration, inadequate training, and ED environmental constraints [13, 14]. Identifying delirium prompts evaluation of reversible precipitants and helps prevent iatrogenic harm. Additional cognitive testing during delirium is often inefficient because dementia screening tests are difficult to interpret. In contrast, many delirium-negative older adults have unrecognized cognitive impairment [6, 12]. Identifying underlying cognitive impairment in these patients informs decision-making capacity, caregiver involvement, communication strategies, and discharge planning [15]. A staged approach may improve feasibility by reserving cognitive impairment screening for delirium-negative patients. Clinicians, therefore, question whether cognitive assessment should extend beyond delirium screening and which brief tools are most practical in the ED [13, 14].

Brief screening tools such as the 4 A's Test (4AT) and brief Confusion Assessment Method (bCAM) for delirium, and the 6-Item Cognitive Impairment Test (6-CIT) and 10-point Cognitive Screener (10-CS) for cognitive impairment have demonstrated accuracy and feasibility in ED settings [10-13, 16]. However, few studies have compared integrated screening strategies with delirium-only or single-test approaches, particularly for post-discharge, patient-centered outcomes. In prior work, we used bCAM followed by 10-CS among delirium-negative patients and identified a broader group of older adults at high risk for in-hospital adverse outcomes [6]. In this study, we compared practical bedside ED cognitive screening strategies (delirium screening alone, cognitive impairment screening alone, and a staged strategy integrating delirium and cognitive impairment screening) to evaluate their predictive value for 90-day functional ADL decline and mortality beyond standard sociodemographic and clinical risk factors among older adults admitted from the ED.

## 2 ∣ Methods

### 2.1 ∣ Study Design, Setting, and Participants

We conducted a prospective cohort study involving adults aged ≥ 65 years admitted from the ED of Hospital Sírio-Libanês, a tertiary academic medical center in São Paulo, Brazil. This study was part of a broader initiative (PRO-AGE) aimed at evaluating geriatric vulnerability and its association with hospital and post-discharge outcomes [17]. Hospital Sírio-Libanês is a high-complexity private institution with 474 beds and 33 specialized centers spanning medical and multidisciplinary care. Since 2017, its ED has hosted a dedicated geriatric emergency care program (PRO-AGE) that provides prioritized, multidisciplinary care for older adults. There are over 90,000 ED visits annually, staffed by onsite geriatricians, interdisciplinary teams, and specialty consultants. The program received Level III Geriatric ED accreditation from the American College of Emergency Physicians in 2019 and 2022 [17].

All patients aged ≥ 65 years who were consecutively admitted from the ED between November 2021 and April 2022 were screened for eligibility. Patients were excluded if they were critically unstable (e.g., required intensive monitoring or urgent procedures), unreachable in the ED within 24 h of admission (e.g., unavailable for assessment due to diagnostic tests or procedures), unable to communicate without assistance, or declined to participate.

The study was approved by the Research Ethics Committee of Hospital Sírio-Libanês. Informed consent was obtained from all participants or their legal representatives [18]. Study data were collected and managed through the Research Electronic Data Capture (REDCap) online platform.

### 2.2 ∣ Data Collection

Trained investigators, mostly registered nurses, conducted a standardized baseline assessment with patients and their representatives in the ED immediately after the decision to admit. Information on sociodemographic characteristics, medical history, baseline frailty and functional status, acute illness severity, and cognitive screening were collected. Sociodemographic variables included age (in years), sex at birth (male or female), self-identified race/ethnicity (categorized as White, Black, or Other), and years of formal education [19]. Comorbidities were assessed from reported information and chart review and summarized using the Charlson Comorbidity Index, a validated measure of overall disease burden [20]. Baseline frailty was assessed using the FRAIL scale (0–5), based on fatigue, resistance, ambulation, illnesses, and unintentional weight loss 4 weeks before the acute illness; scores ≥ 3 indicated frailty [21]. Baseline functional status, based on the patient's status 2–4 weeks before hospitalization, was assessed using the Katz Index (0–6), which evaluates dependence in six basic activities of daily living (ADLs)—bathing, dressing, toileting, transferring, eating, and continence [22, 23]. Acute illness severity was measured using the National Early Warning Score 2 (NEWS-2), a standardized tool that incorporates six physiological parameters recorded at ED admission—respiratory rate, oxygen saturation, temperature, systolic blood pressure, heart rate, and level of consciousness [24, 25]. Investigators also extracted the need for intensive care unit (ICU) admission during hospitalization and length of stay from electronic medical records.

### 2.3 ∣ Cognitive Screening

Cognitive screening was performed using brief bedside instruments to screen for delirium and cognitive impairment.

#### 2.3.1 ∣ Delirium

We administered the brief Confusion Assessment Method (bCAM), a widely validated instrument for detecting delirium in time-constrained acute care settings [26]. The bCAM assesses acute onset and fluctuating course, inattention, disorganized thinking, and altered level of consciousness; a positive result indicates the presence of delirium.

#### 2.3.2 ∣ Cognitive Impairment

We applied the 10-point Cognitive Screener (10-CS), a validated, freely available tool that evaluates temporal orientation, verbal fluency, and three-word recall [27]. The 10-CS takes approximately 2 min to administer, requires no motor or written tasks, and needs no scoring sheet, making it very practical for ED settings [16, 28]. Previous work has shown that 10-CS scores ≤ 5 are indicative of cognitive impairment in acute care settings [6, 16, 28]. This cut-off also showed excellent accuracy for detecting dementia in ambulatory settings and for predicting delirium in acute care settings [16, 27]. In acutely ill older patients, a 10-CS ≤ 5 applied alone does not distinguish between preexisting cognitive impairment and acute cognitive dysfunction, particularly delirium [4, 28]. Administration and scoring instructions for the 10-CS are provided in Figure S1. Additionally, on admission, patients or their proxy answered the Identification of Seniors at Risk (ISAR) memory item, indicating “yes” or “no” to whether they had serious memory problems [29].

#### 2.3.3 ∣ Cognitive Status Classification

**FIGURE 1 ∣:**
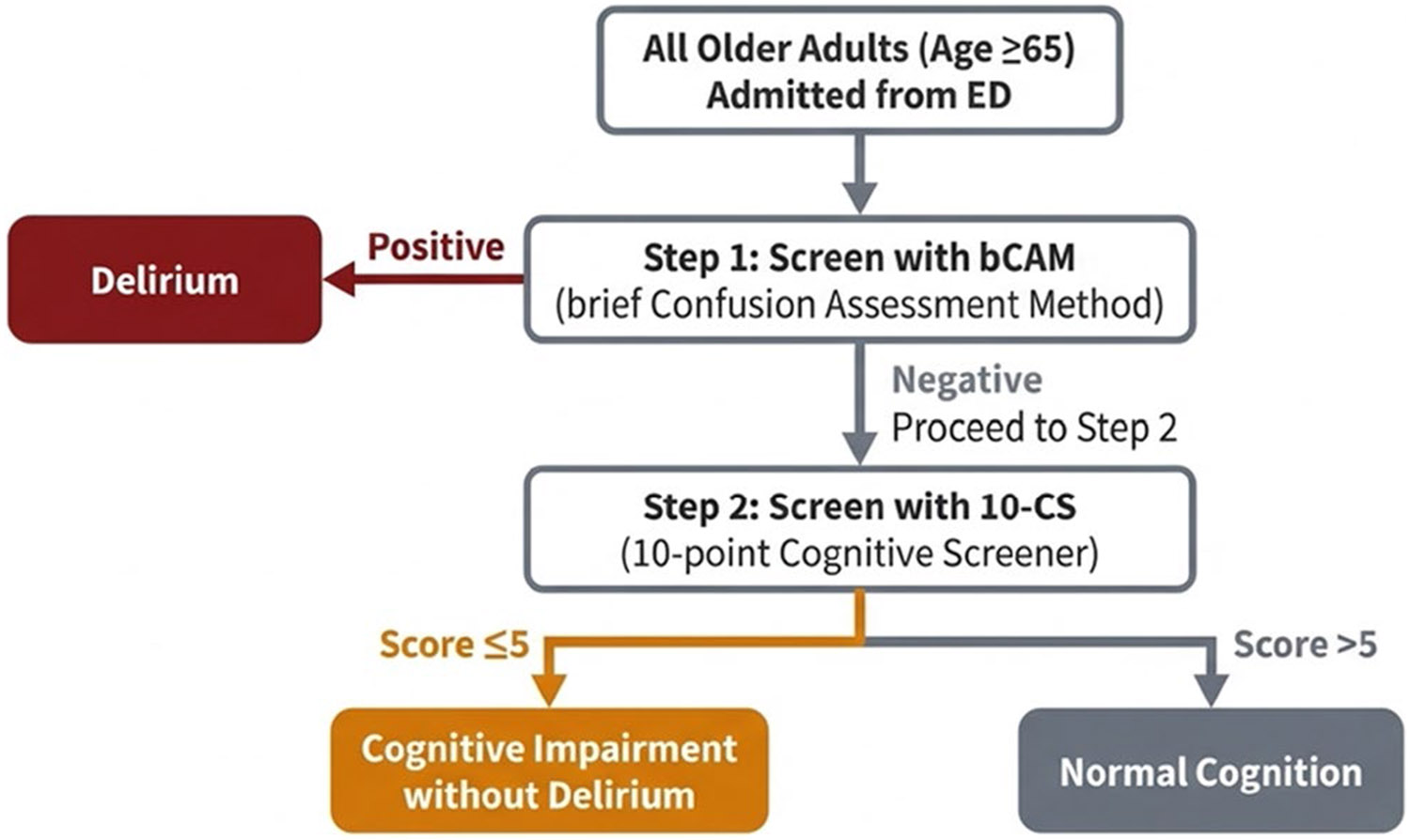
Two-step cognitive screening pathway and cognitive status classification at emergency department admission.

Cognitive status was further operationalized using a two-step pathway based on bCAM and 10-CS results, as shown in Figure 1. Patients were categorized into three groups: (1) normal cognition (bCAM negative and 10-CS > 5), (2) cognitive impairment without delirium (bCAM negative and 10-CS ≤ 5), and (3) delirium (bCAM positive).

### 2.4 ∣ Outcomes

The primary outcomes were time to functional ADL decline and time to death within 90 days of ED admission. Structured telephone interviews were conducted at 30 and 90 days by investigators blinded to baseline assessments. Functional decline was defined as the new need for assistance in performing at least one previously independent ADL—bathing, dressing, toileting, transferring, eating, or continence—relative to the patient's functional status 2–4 weeks before admission [22, 23]. Because the exact timing of new ADL dependence could not be determined, the event date was defined as the midpoint between the two follow-up assessments. For participants who died, the exact date of death was obtained from the hospital electronic health record or, if death occurred after discharge, reported by the proxy during follow-up interviews. Participants or their proxies who were not reached initially were recontacted through up to five additional calls over a 2-week window. This strategy ensured complete follow-up data for all participants. Patients who remained alive and free of functional decline at 90 days were censored [30].

### 2.5 ∣ Statistical Analysis

We reported baseline characteristics using counts and percentages for categorical variables and means and standard deviations (SD) or medians and interquartile ranges (IQR) for continuous variables. Differences across cognitive status groups were tested using chi-squared tests for categorical variables and one-way analysis of variance (ANOVA) or Kruskal–Wallis tests for continuous variables.

We compared discriminative performance for adverse outcomes across three screening strategies using the area under the receiver operating characteristic curve (AUC): a delirium-only strategy based on the bCAM (positive vs. negative), a single-test approach classifying all patients as cognitively impaired or not based on the 10-CS (≤ 5 vs. > 5), and a brief two-step pathway based on bCAM followed by the 10-CS among delirium-negative patients (normal cognition, cognitive impairment without delirium, or delirium).

To evaluate associations between cognitive status and 90-day outcomes, we plotted cumulative incidence function curves for functional ADL decline and Kaplan–Meier curves for mortality. We used Fine–Gray subdistribution hazard models for functional decline (treating death as a competing risk) and Cox proportional hazards models for mortality, reporting subdistribution hazard ratios (sub-HR) and hazard ratios (HR) with 95% confidence intervals (CI) [31]. Models were estimated with and without adjustment for sociodemographic characteristics, Charlson Comorbidity Index, frailty, acute illness severity (NEWS-2), and ICU admission. To avoid overlap with the exposures of interest, we excluded the dementia item from the Charlson Comorbidity Index and the level-of-consciousness item from NEWS-2 [25].

We used continuous net reclassification improvement (NRI) to assess the predictive value of sequentially adding delirium status (bCAM) and cognitive impairment (10-CS) to nested models. NRI quantifies the net proportion of participants with events reassigned to higher predicted risk plus the net proportion without events reassigned to lower risk; the overall NRI is the sum of these two components, with values of 0.20, 0.40, and 0.60 reflecting small, moderate, and large improvements, respectively [32]. We added bCAM (positive vs. negative) to a prespecified base model of sociodemographic and clinical variables and then added 10-CS (≤ 5 vs. > 5) to the base model plus bCAM.

Supplementary analyses evaluated the single-test 10-CS approach in prediction models. Sensitivity analyses examined whether classifying delirium-negative patients as having cognitive impairment based on a documented dementia diagnosis or reported serious memory problems could serve as a pragmatic alternative to the 10-CS; agreement between methods was assessed using the kappa statistic.

All statistical tests were two-sided, with statistical significance set at *p* < 0.05. Analyses were conducted as complete case analyses using Stata 17 (StataCorp, College Station, TX, USA).

## 3 ∣ Results

During the 6-month study period, 1178 patients were admitted from the ED. After excluding readmissions of patients already enrolled (*n* = 178), patients who were unable to communicate without a proxy (*n* = 11), clinically unstable (*n* = 33), required urgent procedures (*n* = 40), were unreachable by the research team (*n* = 41), and declined participation (*n* = 45), the final sample comprised 830 older adults (Figure S2). Participants had a mean (SD) age of 79.7 (8.7) years; 46.6% were women, and 91.8% self-identified as White. The median educational attainment was 15 years (IQR = 11–17). In total, 35.2% of participants were classified as frail, 18.9% had a previous diagnosis of dementia (chart-documented or proxy-reported), and 38.5% reported serious memory complaints (Table 1). The median hospital length of stay was 5.2 days (IQR = 2.8–9.2), and 5.9% required ICU admission.

Using the two-step pathway based on bCAM and 10-CS results, we classified 427 (51.5%) patients as having normal cognition, 171 (20.6%) as having delirium, and 232 (27.9%) as having cognitive impairment without delirium. Compared to patients with normal cognition, those with cognitive impairment, regardless of delirium status, were generally older, more often female, had fewer years of formal education, greater comorbidity burden, increased frailty, greater baseline dependence in ADLs, and more severe acute illness scores (Table 1). Patients with cognitive impairment, regardless of dementia status, also had longer hospital stays. Notably, 52.2% of patients without delirium but with cognitive impairment had neither a prior documented dementia diagnosis nor reported serious memory problems, compared to 12.9% of those with delirium.

Within 90 days, 142 patients (22.2%) experienced functional ADL decline and 74 (11.1%) died—38 during hospitalization and 36 after discharge. For 90-day functional decline, the brief two-step pathway showed higher discrimination (AUC = 0.68, 95% CI = 0.63–0.73) than delirium alone (AUC = 0.61, 95% CI = 0.57–0.65) and the single-test 10-CS approach (AUC = 0.65, 95% CI = 0.61–0.70), with both pairwise comparisons *p* < 0.001. For 90-day mortality, the brief two-step pathway (AUC = 0.70, 95% CI = 0.64–0.76) also outperformed delirium alone (AUC = 0.64, 95% CI = 0.58–0.70) and the single-test 10-CS approach (AUC = 0.66, 95% CI = 0.61–0.71), with pairwise comparisons *p* = 0.002 and *p* = 0.008, respectively.

**FIGURE 2 ∣:**
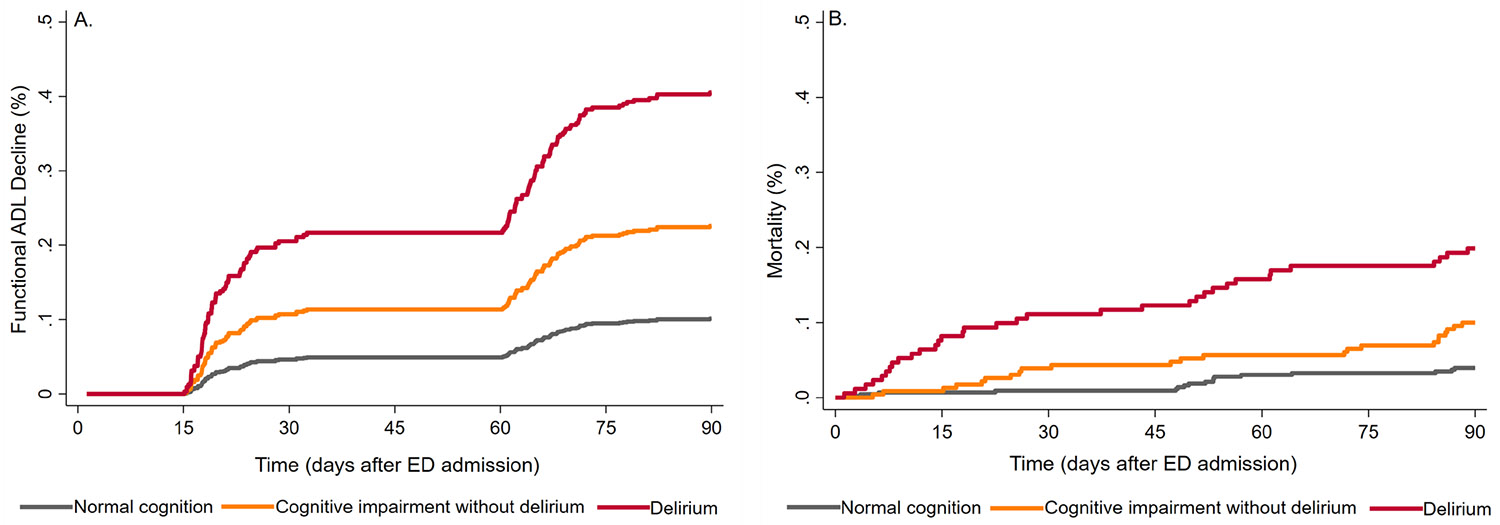
Cumulative incidence of adverse outcomes based on cognitive status at emergency department admission: (A) functional ADL decline (*n* = 769) and (B) mortality (*n* = 830). Functional ADL decline was defined as new dependence in one or more ADL within 90 days of the ED visit, compared to 2–4 weeks prior to admission (61 patients who were fully dependent before ED admission were excluded from this analysis). ADL = basic activities of daily living (bathing, dressing, toileting, transferring, eating, and continence).

Compared with patients with normal cognition, those with delirium or without delirium but with cognitive impairment had a higher cumulative incidence of functional ADL decline and mortality throughout follow-up after ED admission (Figure 2). At 90 days, the cumulative incidence of functional ADL decline increased across cognitive status groups (10.3% with normal cognition, 23.5% without delirium but with cognitive impairment, and 45.0% with delirium; *p* < 0.001). Similarly, 90-day mortality showed a graded increase (3.7%, 10.3%, and 19.9% across the same three groups, respectively; *p* < 0.001), as shown in Table 2. After adjusting for sociodemographic and clinical measures, patients without delirium but with cognitive impairment had a higher risk of 90-day functional ADL decline and mortality than those with normal cognition. These risks were similar to those observed among patients with delirium (Table 2).

Adding delirium status (bCAM) to the prespecified base model of sociodemographic and clinical variables produced a small-to-moderate improvement in 90-day risk reclassification for mortality, but no significant improvement for functional ADL decline (Table 3). When cognitive impairment (10-CS) was added to the base model plus bCAM, risk reclassification for both adverse outcomes improved, with a moderate improvement for functional ADL decline and an additional small-to-moderate improvement for mortality.

In supplementary analyses using the single-test 10-CS approach, cognitive impairment (10-CS ≤ 5) was associated with a higher risk of 90-day functional ADL decline (adjusted sub-HR = 1.82; 95% CI = 1.19–2.79) and 90-day mortality (adjusted HR = 2.57; 95% CI = 1.38–4.76) compared with 10-CS > 5 (Table S1).

Among delirium-negative patients, agreement between 10-CS–defined cognitive impairment (≤ 5) and the pragmatic alternative definition (documented dementia diagnosis or reported serious memory problems) was 70.3%, indicating fair agreement (kappa = 0.32; 95% CI: 0.24–0.39). Using this pragmatic classification, delirium and cognitive impairment without delirium remained associated with higher 90-day mortality compared with normal cognition, but cognitive impairment without delirium was not associated with functional ADL decline after adjustment (Table S2).

## 4 ∣ Discussion

This study demonstrated that brief cognitive screening at ED admission can identify older adults hospitalized from the ED who are at increased risk of 90-day functional ADL decline and mortality. Older patients with delirium and those without delirium but with cognitive impairment had similarly elevated risks for adverse outcomes. More than half of delirium-negative patients classified as having cognitive impairment had no documented dementia diagnosis or reported serious memory problems. In comparative analyses, a bedside strategy integrating delirium assessment with cognitive impairment evaluation among delirium-negative patients showed higher discrimination for adverse outcomes than delirium-only screening or a single-test strategy.

The American Geriatrics Society's Geriatric Emergency Department Guidelines recommend routine cognitive screening in the ED to identify delirium and baseline cognitive impairment, given their high prevalence and clinical impact [9, 33]. Nearly half of our cohort screened positive for cognitive impairment—21% with delirium and 28% without delirium but with cognitive impairment. This prevalence is higher than estimates from broader ED cohorts but likely reflects our older, higher-acuity admitted sample (mean age 80 years) [3, 12, 34]. Cognitive impairment affects approximately 28% to 38% of older ED patients (exceeding 50% among those aged ≥ 85 years), and delirium is identified in about 15% of geriatric ED presentations. Dementia and milder cognitive impairment remain frequently overlooked, with 55% to 60% of cognitive disorders in acute care going unrecognized [9, 29]. In our study, over half of patients without delirium but with cognitive impairment lacked a documented dementia diagnosis or reported serious memory problems. This gap is clinically important because identifying underlying cognitive impairment may affect decision-making capacity assessment, caregiver engagement, communication strategies, and discharge planning [15].

Few studies have implemented a structured, sequential approach to screen for delirium, followed by cognitive impairment assessment in patients without delirium at ED admission. Most existing research has assessed these conditions separately or used tools like the 4AT, which screen for both but do not distinguish between them [10, 11]. Performance-based cognitive tests, such as the Short Blessed Test (SBT), Brief Alzheimer's Screen (BAS), and Ottawa 3DY (O3DY), have been used in ED settings but are rarely paired with validated delirium assessments [34]. The Eight-item Ascertain Dementia Screening Interview (AD8) and the 16-item Informant Questionnaire on Cognitive Decline in the Elderly (IQCODE-16) have also been evaluated, but both rely on a knowledgeable informant, which is often a limiting factor in ED settings [5]. In previous studies using combined screening, the 4AT and 6-CIT identified delirium in 15% and dementia in 21% of older adults [12]. The higher prevalence observed in our study may partly reflect the 10-CS's diagnostic performance compared to the 6-CIT [27]. A large single-center study in Scotland reported rates similar to ours but used a more time-consuming dual cognitive screening approach, which may be less feasible in busy ED settings [14, 35].

Delirium has emerged as a powerful predictor for identifying high-risk acutely ill older adults in the ED [36-38]. The incremental prognostic value of underlying cognitive impairment, particularly for longer-term, patient-centered outcomes such as functional decline, is less well defined [4]. Cognitive impairment identified at ED presentation has been associated with higher risks of death, institutionalization, and functional decline over 3 to 12 months, although many studies did not account for delirium [39, 40]. In our data, a single-test 10-CS strategy applied to all patients was also associated with adverse outcomes but showed lower discrimination than a staged strategy, which distinguished delirium from cognitive impairment without delirium. In prior work, delirium-negative patients with cognitive impairment had in-hospital risks comparable to those with delirium [6]. Extending these findings, we observed that delirium and delirium-negative cognitive impairment were each associated with higher 90-day risks of functional decline and mortality. Adding cognitive impairment to delirium status improved risk reclassification for both outcomes, whereas delirium alone improved reclassification only for mortality.

Our findings have practical implications for acute care settings. Distinguishing delirium from underlying cognitive impairment is clinically important. Delirium is a medical emergency that requires urgent evaluation of reversible precipitants. Underlying cognitive impairment informs communication strategies, capacity assessment, caregiver engagement, and discharge planning. By first screening for delirium with the bCAM and then administering the 2-min 10-CS to delirium-negative patients, this staged strategy minimizes testing burden during delirium while detecting clinically meaningful cognitive vulnerability [6, 41]. This early identification can inform decisions such as conducting comprehensive geriatric assessments, avoiding anticholinergic medications, and prioritizing continuity of care and post-discharge follow-up [42, 43]. A pragmatic definition based on documented dementia or serious memory problems may capture mortality risk but overlook subtler impairments relevant to functional decline. When brief cognitive testing is not feasible, this approach offers a low-burden alternative for busy settings, although formal cognitive assessment remains preferable when possible [44-46]. Structured screening strategies may become increasingly relevant as emergency departments care for a growing number of older adults with cognitive vulnerability. The anticipated availability of blood-based dementia tests may further increase ED visits related to cognitive concerns [47, 48].

Our results should be considered in light of methodological limitations. This was a single-center study conducted at a high-complexity, geriatric-accredited private hospital, which may limit generalizability and feasibility in less-resourced ED settings. Functional outcomes were assessed by telephone, including self- and proxy-reports, which may have introduced information bias. However, this approach enabled complete follow-up and reflects common post-discharge monitoring practices [49]. Moreover, any non-differential misclassification would likely bias our results toward the null. Our screening strategy did not distinguish delirium superimposed on dementia from delirium alone. Cognitive impairment without delirium may reflect preexisting cognitive impairment, although acute contributors cannot be fully excluded. Nevertheless, in the ED, the aim is to identify cognitive impairment rather than to diagnose underlying dementia. Cognitive impairment, with or without delirium, should prompt consideration of comprehensive geriatric assessment and cognitive follow-up [50]. We also did not capture new or persistent delirium episodes after ED admission, as our focus was on early recognition of cognitive vulnerability at the frontline. Because incident delirium is strongly linked to underlying cognitive impairment, our dual-screening approach might have partially captured its associated risk [28]. Additionally, caution is warranted when extrapolating to other cognitive screening tools, as we did not test dual screening using other brief assessments.

The study also has key strengths. We tested a pragmatic and efficient dual cognitive screening approach using brief, validated bedside tools that require minimal resources and no collateral history. Follow-up assessments were conducted by blinded investigators and included longer-term, patient-centered outcomes such as functional status, which are clinically meaningful but often underreported in the literature. We also applied rigorous statistical methods, including competing risk analyses and models incorporating standard acute-care risk factors [31]. Still, multicenter studies are needed to evaluate implementation and equity implications in diverse ED settings and to test whether cognitive screening, extended to patients discharged home, improves care processes, transitions, and patient-centered outcomes [13].

In conclusion, this study demonstrated that brief cognitive screening at ED admission identified hospitalized older adults at increased risk for adverse outcomes. Integrating delirium assessment with cognitive impairment screening among delirium-negative patients showed higher discrimination for adverse outcomes than delirium screening alone. This practical two-step strategy may enhance the early recognition of cognitive vulnerability, support risk stratification, and guide more personalized care for older adults admitted from the ED.

## Supplementary Material

jgs70378-sup-0001-supinfo

Additional supporting information can be found online in the Supporting Information section. **Figure S1:** Administration and scoring guidance for the 10-point Cognitive Screener (10-CS). **Figure S2:** Flowchart of the study participants. **Table S1:** Association of cognitive impairment defined by the 10-point Cognitive Screener with 90-day functional decline and mortality. **Table S2:** Association of a pragmatic cognitive classification strategy with 90-day functional ADL decline and mortality.

## Figures and Tables

**TABLE 1 ∣:** Characteristics of older ED patients according to cognitive status.

Variables	Total (*n* = 830)	Normal	Cognitive Impairment		*p*
		cognition	No delirium	Delirium	
		(*n* = 427)	(*n* = 232)	(*n* = 171)	
Sociodemographic factors					
Age (years), mean (SD)	79.7 (8.7)	75.9 (7.0)	81.9 (8.5)	86.0 (7.8)	< 0.001
Female sex, *n* (%)	387 (46.6)	176 (41.2)	118 (50.9)	93 (54.4)	0.004
White race/ethnicity, *n* (%)	762 (91.8)	394 (92.3)	211 (90.9)	157 (91.8)	0.84
Education (years), median (IQR)	15 (11, 17)	16 (11, 17)	14 (11, 17)	13 (11, 16)	< 0.001
Clinical characteristics					
Charlson comorbidity index, median (IQR)	1 (0; 2)	1 (0; 2)	1 (0; 3)	2 (1; 3)	0.001
Memory problems and/or dementia diagnosis, *n* (%)	335 (40.4)	75 (17.6)	111 (47.8)	149 (87.1)	< 0.001
Frail status (FRAIL scale ≥ 3), *n* (%)	292 (35.2)	87 (20.4)	87 (37.5)	118 (69.0)	< 0.001
Katz ADL Index (0–6), median (IQR)	0 (0, 3)	0 (0, 0)	1 (0, 3)	5 (3, 6)	< 0.001
Acute illness severity (NEWS-2), median (IQR)	1 (0, 3)	1 (0, 2)	1 (0, 2)	2 (0, 3)	< 0.001
Hospital-related measures					
ICU admission, *n* (%)	49 (5.9)	23 (5.4)	17 (7.3)	9 (5.3)	0.55
Length of stay (days), median (IQR)	5.2 (2.8, 9.2)	3.9 (2.1, 7.3)	6.0 (3.2, 10.1)	8.3 (4.9, 15.7)	< 0.001

*Note:* Cognitive status was operationalized based on the brief Confusion Assessment Method (bCAM) and the 10-point Cognitive Screener (10-CS): normal cognition (bCAM negative and 10-CS > 5), delirium (bCAM positive), or no delirium with cognitive impairment (bCAM negative and 10-CS ≤ 5). For analytical purposes, we used modified scoring algorithms for NEWS-2 (excluding the consciousness component) and the Charlson Comorbidity Index (excluding the dementia item) to reduce overlap with delirium and cognitive impairment. Katz ADL Index (0–6), assessed on admission based on the patient’s functional status 2–4 weeks prior hospitalization, with higher scores indicating greater dependence in ADL. Comparisons investigated differences among the three cognitive status groups using the one-way analysis of variance (ANOVA) test (normal distribution) or the Kruskal–Wallis test (non-normal distribution) for continuous variables and the chi-squared test for categorical variables.

Abbreviations: ADL = activities of daily living (bathing, dressing, toileting, transferring, eating, and continence); ED = emergency department; ICU = intensive care unit; NEWS-2 = national early warning score 2; QR = interquartile range; SD = standard deviation.

**TABLE 2 ∣:** Association between cognitive status at the emergency department admission and adverse outcomes within 90 days.

Outcomes	*N* events/*N* total (%)	Sub-HR or HR (95% confidence interval)	
		Unadjusted	Adjusted
Functional ADL decline (*n* = 769)			
Normal cognition	43/425 (10.3)	(reference)	(reference)
Cognitive impairment without delirium	51/226 (23.5)	2.38 (1.59–3.58)	1.60 (1.03–2.49)
Delirium	48/118 (45.0)	4.86 (3.22–7.34)	2.41 (1.37–4.26)
Mortality (*n* = 830)			
Normal cognition	16/427 (3.7)	(reference)	(reference)
Cognitive impairment without delirium	24/232 (10.3)	2.84 (1.51–5.34)	2.31 (1.18–4.51)
Delirium	34/171 (19.9)	5.90 (3.26–10.69)	2.96 (1.48–5.92)

*Note:* Estimates were computed from Fine–Gray models for functional ADL decline, considering death as a competing risk, and Cox proportional hazards models for mortality. Functional ADL decline was defined as new dependence in ≥ 1 ADL within 90 days of ED admission, compared to the 2–4 weeks prior to admission (61 patients who were fully dependent before ED admission were excluded from this analysis). Percentages represent the 90-day cumulative incidence of each outcome. Cognitive status was operationalized based on the brief Confusion Assessment Method (bCAM) and the 10-point Cognitive Screener (10-CS): normal cognition (bCAM negative and 10-CS > 5), delirium (bCAM positive), or no delirium with cognitive impairment (bCAM negative and 10-CS ≤ 5). Adjusted models included sociodemographic characteristics (age, sex, race/ethnicity, and education), the Charlson Comorbidity Index (excluding dementia), frailty, the National Early Warning Score 2 (NEWS-2, excluding the consciousness item), and intensive care unit admission. There were no significant differences between patients with delirium and those without delirium but with cognitive impairment in the adjusted models for 90-day functional ADL decline (sub-HR 1.51; 95% CI 0.95–2.39; *p* = 0.08), or for 90-day mortality (HR 1.28; 95% CI 0.74–2.24; *p* = 0.38).

**TABLE 3 ∣:** Incremental value of sequential cognitive screening measures for 90-day adverse outcome risk reclassification.

Outcomes	Continuous net reclassification improvement (NRI)							
	bCAM added to base model				10-CS added to base model + bCAM			
	For events	For nonevents	Overall (95% CI)	*p* ^a^	For events	For nonevents	Overall (95% CI)	*p* ^a^
Functional ADL decline (*n* = 769)	−0.27	0.43	0.16 (−0.02–0.34)	0.08	0.07	0.34	0.41 (0.23–0.59)	< 0.001
Mortality (*n* = 830)	0.00	0.28	0.28 (0.04–0.51)	0.02	0.00	0.26	0.26 (0.02–0.50)	0.03

*Note:* Functional ADL decline was defined as new dependence in ≥ 1 ADL within 90 days of ED admission, compared to the 2–4 weeks prior to admission (61 patients who were fully dependent before ED admission were excluded from this analysis). Delirium status (bCAM positive vs. negative) was added to a prespecified base model including sociodemographic characteristics (age, sex, race/ethnicity, and education), the Charlson Comorbidity Index (excluding dementia), frailty, the National Early Warning Score 2 (NEWS-2, excluding the consciousness item), and intensive care unit admission. Cognitive impairment (10-CS ≤ 5 vs. > 5) was then added to the base model plus bCAM.

Abbreviations: 10-CS = 10-point Cognitive Screener (scores ≤ 5 define cognitive impairment); 95% CI = 95% confidence interval; ADL = basic activities of daily living (bathing, dressing, toileting, transferring, eating, and continence); bCAM = brief Confusion Assessment Method.

a *p* values correspond to the continuous NRI, which quantifies the net correct movement in predicted risk (upward for patients with events and downward for those without).

## Summary

### Key points

- Among older adults admitted from the emergency department, 49% had cognitive impairment, including 21% with delirium and 28% without, and over half of delirium-negative patients with cognitive impairment had no documented dementia diagnosis or reported memory problems.
- Similar to delirium, cognitive impairment without delirium was associated with higher risks of 90-day functional decline and mortality compared with normal cognition, even after adjustment for standard sociodemographic and clinical factors.
- A brief staged strategy that screened for delirium followed by cognitive testing in delirium-negative patients showed better discrimination for 90-day functional decline and mortality than delirium-only or single-test cognitive screening.

### Why does this paper matter?

- Cognitive impairment, including delirium, affects nearly half of older adults admitted from the emergency department but is often missed without systematic screening.
- A brief staged approach—using the Confusion Assessment Method followed by the 10-point Cognitive Screener in delirium-negative patients—showed better discrimination for 90-day functional decline and mortality, offering a practical pathway to identify cognitive vulnerability and guide targeted acute care.
