# Supplementary material for: Two Brief Steps, Better Foresight: Cognitive Screening and Adverse Outcomes in Older Adults Admitted From the Emergency Department
Source: J Am Geriatr Soc. Author manuscript; Available in PMC 2026 May 8. (PMC13150744; doi:10.1111/jgs.70378)
Supplement: jgs70378-sup-0001-supinfo [file NIHMS2165658-supplement-jgs70378-sup-0001-supinfo.pdf]

Two brief steps, better foresight: Cognitive screening and adverse outcomes in older adults admitted from the emergency department

This supplementary material for online-only contains:

Supplementary Figure S1. Administration and scoring guidance for the 10-point Cognitive Screener (10-CS)

Supplementary Figure S2. Flowchart of the study participants

Supplementary Table S1. Association of cognitive impairment defined by the 10-point Cognitive Screener with 90-day functional decline and mortality

Supplementary Table S2. Association of a pragmatic cognitive classification strategy with 90-day functional ADL decline and mortality

ORIENTATION

What year is this?	**0**	**1**
What month is this?	**0**	**1**
What is today's date?	**0**	**1**

(consider only exact answers as correct)

LEARNING

Now I am going to name three objects. Please wait until I say the three objects and then repeat. Remember what they are. I am going to ask you to name them again in a few minutes.

(up to three trials)

VERSION A	VERSION B	VERSION C	
glasses	hat	clock	No points
pen	coin	key	
hammer	flashlight	broom	

VERBAL FLUENCY

Now tell me the names of all animals you can think of, as quick as possible.

I'll give you one minute to come up with as many animals as you can. Ready?

1. ______	11. ______	21. ______
2. ______	12. ______	22. ______
3. ______	13. ______	23. ______
4. ______	14. ______	24. ______
5. ______	15. ______	25. ______
6. ______	16. ______	26. ______
7. ______	17. ______	27. ______
8. ______	18. ______	28. ______
9. ______	19. ______	29. ______
10. ______	20. ______	30. ______

ANIMALS	
0-5	**0**
6-8	**1**
9-11	**2**
12-14	**3**
≥ 15	**4**

RECALL

Now what were the three objects I asked you to remember?

VERSION A	VERSION B	VERSION C		
glasses	hat	clock	**0**	**1**
pen	coin	key	**0**	**1**
hammer	flashlight	broom	**0**	**1**

Adjustment for education effects (10-CS-Edu)

- No formal education: Add 2 points (to a max of 10)
- 1-3 years of education: Add 1 point (to a max of 10)

Interpretation of the 10-CS-Edu

- ≥ 8 points: **Normal**
- 6-7 points: **Possible Cognitive Impairment** (usually compatible with mild cognitive impairment)
- 0-5 points: **Probable Cognitive Impairment** (usually compatible with dementia)

10-CS: ________

10-CS-Edu: ________

Supplementary Figure S1. Administration and scoring guidance for the 10-point Cognitive Screener (10-CS)

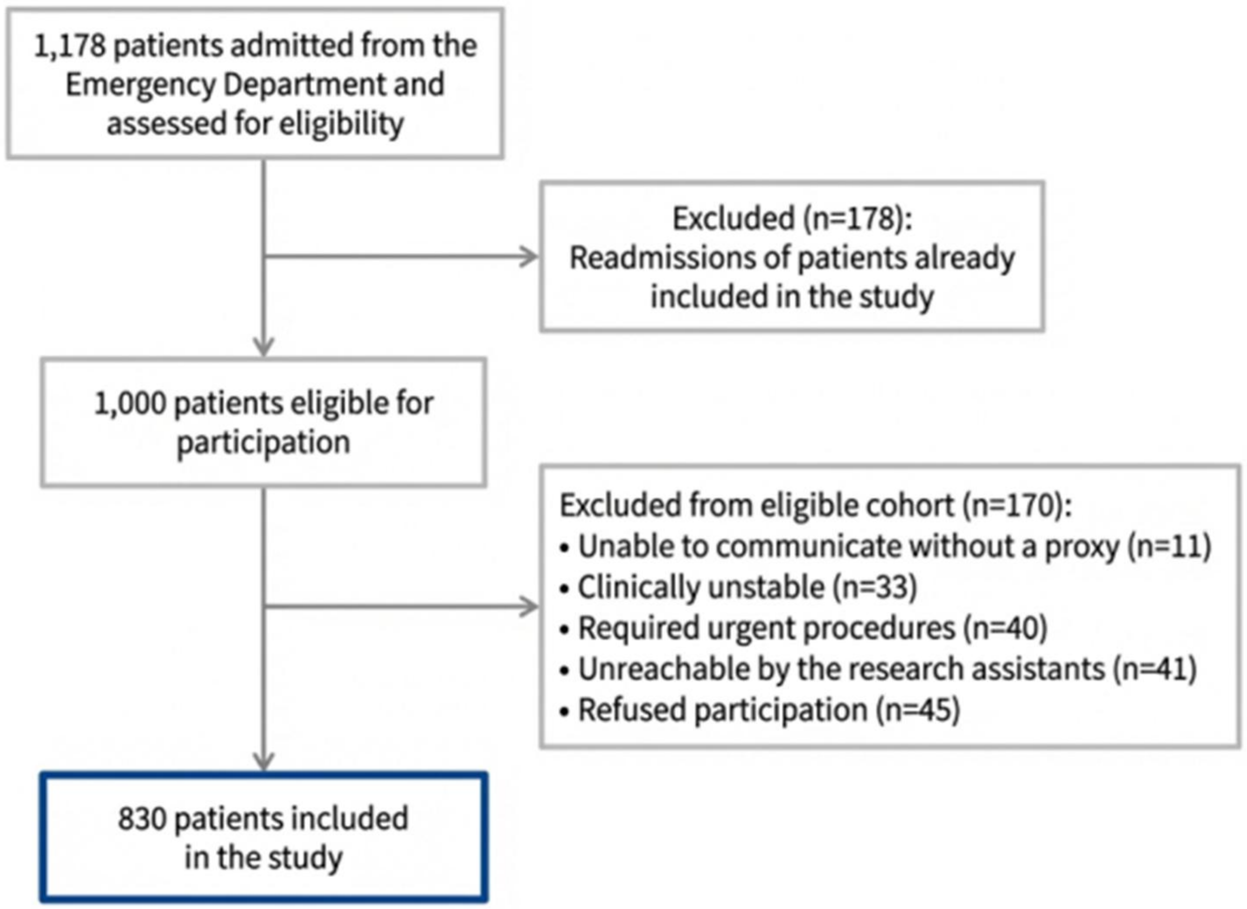

Supplementary Figure S2. Flowchart of the study participants

Supplementary Table S1. Association of cognitive impairment defined by the 10-point Cognitive Screener (10-CS) with 90-day functional decline and mortality

	N events / N total (%)	Sub-HR or HR (95% CI)	
Outcomes		**Unadjusted**	**Adjusted**
Functional ADL decline			
Cognitive impairment			
No (10-CS > 5)	43/425 (10.3)	(reference)	(reference)
Yes (10-CS ≤ 5)	99/344 (30.5)	3.16 (2.21–4.53)	1.82 (1.19–2.79)
Mortality			
Cognitive impairment			
No (10-CS > 5)	16/427 (3.7)	(reference)	(reference)
Yes (10-CS ≤ 5)	58/403 (14.4)	4.08 (2.34–7.09)	2.57 (1.38–4.76)

HR = hazard ratio; ADL = basic activities of daily living (bathing, dressing, toileting, transferring, eating, and continence); ED = emergency department; 95% CI = 95% confidence interval.

Estimates were computed from Fine–Gray models for functional ADL decline, considering death as a competing risk, and Cox proportional hazards models for mortality. Functional ADL decline was defined as new dependence in ≥1 ADL within 90 days of ED admission, compared to the 2–4 weeks prior to admission (61 patients who were fully dependent before ED admission were excluded from this analysis).

Adjusted models included sociodemographic characteristics (age, sex, race/ethnicity, and education), the Charlson Comorbidity Index (excluding dementia), frailty, the National Early Warning Score 2 (NEWS-2, excluding the consciousness item), and intensive care unit admission.

Supplementary Table S2. Association of a pragmatic cognitive classification strategy with 90-day functional ADL decline and mortality

	N events / N total (%)	Sub-HR or HR (95% confidence interval)	
Outcomes		**Unadjusted**	**Adjusted**
Functional ADL decline (n=769)			
Normal cognition	59/470 (12.8)	(reference)	(reference)
Cognitive impairment without delirium	35/181 (20.1)	1.61 (1.06–2.44)	1.19 (0.77–1.84)
Delirium	48/118 (45.0)	3.87 (2.65–5.67)	1.93 (1.15–3.23)
Mortality (n=830)			
Normal cognition	21/473 (4.4)	(reference)	(reference)
Cognitive impairment without delirium	19/186 (10.2)	2.36 (1.27–4.39)	2.17 (1.14–4.14)
Delirium	34/171 (19.9)	4.97 (2.88–8.56)	2.53 (1.35–4.74)

HR = hazard ratio; sub-HR = subdistribution hazard ratio; ADL = basic activities of daily living (bathing, dressing, toileting, transferring, eating, and continence); ED = emergency department.
Estimates were computed from Fine–Gray models for functional ADL decline, considering death as a competing risk, and Cox proportional hazards models for mortality. Functional ADL decline was defined as new dependence in ≥1 ADL within 90 days of ED admission, compared to the 2–4 weeks prior to admission (61 patients who were fully dependent before ED admission were excluded from this analysis). Percentages represent the 90-day cumulative incidence of each outcome.
Cognitive status was classified using a pragmatic strategy based on the brief Confusion Assessment Method (bCAM) and documented dementia diagnosis or reported serious memory problems: normal cognition (bCAM negative and no documented dementia diagnosis or reported serious memory problems), cognitive impairment without delirium (bCAM negative with documented dementia diagnosis or reported serious memory problems), and delirium (bCAM positive).
Adjusted models included sociodemographic characteristics (age, sex, race/ethnicity, and education), the Charlson Comorbidity Index (excluding dementia), frailty, the National Early Warning Score 2 (NEWS-2, excluding the consciousness item), and intensive care unit admission.
There were no significant differences between patients with delirium and those without delirium but with cognitive impairment in the adjusted models for 90-day functional ADL decline (sub-HR 1.62; 95% CI 0.98–2.70; p=0.06) or for 90-day mortality (HR 1.16; 95% CI 0.64–2.12; p=0.62).
